# Central and Peripheral Thermal Signatures of Brain-Derived Fatigue during Unilateral Resistance Exercise: A Preliminary Study

**DOI:** 10.3390/biology11020322

**Published:** 2022-02-17

**Authors:** David Perpetuini, Damiano Formenti, Pierpaolo Iodice, Daniela Cardone, Chiara Filippini, Antonio Maria Chiarelli, Giovanni Michielon, Athos Trecroci, Giampietro Alberti, Arcangelo Merla

**Affiliations:** 1 Department of Neuroscience and Imaging, Institute for Advanced Biomedical Technologies, University G. D’Annunzio of Chieti-Pescara, Via Luigi Polacchi 13, 66100 Chieti, Italy; david.perpetuini@unich.it (D.P.); d.cardone@unich.it (D.C.); chiara.filippini@unich.it (C.F.); antonio.chiarelli@unich.it (A.M.C.); arcangelo.merla@unich.it (A.M.); 2 Department of Biotechnology and Life Sciences (DBSV), University of Insubria, Via Dunant, 3, 21100 Varese, Italy; 3 Center for the Study and the Transformation of Physical Activities, Faculty of Sport Sciences, University of Rouen Normandie, Rue Thomas Becket, 76130 Rouen, France; pierpaolo.iodice@univ-rouen.fr; 4 Department of Biomedical Sciences for Health, University of Milan, Via Kramer 4, 20129 Milan, Italy; giovanni.michielon@unimi.it (G.M.); athos.trecroci@unimi.it (A.T.); giampietro.alberti@unimi.it (G.A.)

**Keywords:** infrared thermography, thermal imaging, strength training, resistance training, unilateral exercise, frequency-domain analysis

## Abstract

**Simple Summary:**

Fatigue is considered a brain-derived emotion that could impact performance during the execution of physical exercises. Infrared thermography is a valuable technique able to measure the psychophysiological state associated with emotions in a contactless manner. The aim of the study is to test the capability of infrared thermography to evaluate the central and peripheral physiological effect of fatigue through facial skin and muscle temperature modulations collected during the execution of a unilateral resistance exercise of the lower limb. Both time- and frequency-domain analyses were performed on the temperature time course of the face and limbs. Particularly, significant correlations between features extracted from the thermal signals and the perceived exertion were found. These findings confirmed the ability of thermal imaging to detect both peripheral and central effects of fatigue in response to physical exercises. These results could foster the employment of infrared thermography to monitor the psychophysiological state of the athletes during training. The possibility to calibrate the training load in accordance with the psychophysiological conditions could improve the performance of the athletes during the training process and competitions.

**Abstract:**

Infrared thermography (IRT) allows to evaluate the psychophysiological state associated with emotions from facial temperature modulations. As fatigue is a brain-derived emotion, it is possible to hypothesize that facial temperature could provide information regarding the fatigue related to exercise. The aim of this study was to investigate the capability of IRT to assess the central and peripheral physiological effect of fatigue by measuring facial skin and muscle temperature modulations in response to a unilateral knee extension exercise until exhaustion. Rate of perceived exertion (RPE) was recorded at the end of the exercise. Both time- (∆T_ROI_: pre–post exercise temperature variation) and frequency-domain (∆PSD: pre–post exercise power spectral density variation of specific frequency bands) analyses were performed to extract features from regions of interest (ROIs) positioned on the exercised and nonexercised leg, nose tip, and corrugator. The ANOVA-RM revealed a significant difference between ∆T_ROI_ (F_(1.41,9.81)_ = 15.14; *p* = 0.0018), and between ∆PSD of myogenic (F_(1.34,9.39)_ = 15.20; *p* = 0.0021) and neurogenic bands (F_(1.75,12.26)_ = 9.96; *p* = 0.0034) of different ROIs. Moreover, significant correlations between thermal features and RPE were found. These findings suggest that IRT could assess both peripheral and central responses to physical exercise. Its applicability in monitoring the psychophysiological responses to exercise should be further explored

## 1. Introduction

Infrared thermography (IRT) is a technology able to measure superficial temperatures in a contactless manner [1]. This technique has been demonstrated to be informative of the psychophysiological state of the subject by detecting thermal modulations usually extracted from responsive regions (e.g., nose tip, corrugator, perioral regions) [2], allowing us to identify stress, anxiety, emotions, and fatigue [3,4,5,6,7]. Skin temperature changes through thermal imaging are generally evaluated by means of time- and frequency-domain-based analyses [8,9].

Thanks to its contactless features, IRT may be highly suited in applications where the ecological setting has to be preserved, such as clinical applications and physical exercise [8,10,11,12]. Particularly, thermal imaging can provide information regarding the superficial circulation of muscles, indicative of thermoregulation processes induced by physical exercise [13]. In fact, during exercise, body compensatory vasoregulation mechanisms are activated through reduction in blood flow in the splanchnic region and tegumentary apparatus [12,14]. Particularly, excess heat should be transported from the core to the skin and then transferred to the environment [15]. At rest, heat is actually transported from the core to skeletal muscle tissue, whereas during exercise, the increased muscle temperature provokes the inversion of the temperature gradient between muscle and arterial blood. Hence, during exercise, heat is transferred from muscle to blood and subsequently to the body core, favouring an increase in the venous return and the cardiac output. The ability to modulate skin blood flow, and consequently superficial temperature, constitutes a powerful defence mechanism against hyperthermia [15].

Intense exercise induces also physical fatigue, which is predominantly considered a brain-derived emotion, part of a complex regulation system that aims to protect the whole-body homeostasis [16,17]. Since emotions lead to facial skin temperature modifications [18,19], IRT may be used to provide information on the fatigue state of the subjects while performing exercises [3]. Perception of fatigue is unique to each individual and it is commonly measured by means of questionnaires (e.g., the Borg rating of perceived exertion, RPE) [20]. RPE allows to measure physical exercise intensity, relying on how hard the subject perceives the exercise to be while performing it. It is based on the physical sensations that an individual undergoes during exercise. It was demonstrated that RPE correlates with heart rate [21], proving its relationship with physiological changes of the body associated with fatigue. In this sense, IRT may be a suitable technique to provide information regarding the perception of fatigue based on physiological adaptation to exercise. Although sensation of fatigue has been extensively investigated with thermal imaging in response to cognitive tasks (i.e., mental fatigue) [3,22], to the best of the authors’ knowledge, there is a paucity of literature investigating the thermal signature of fatigue in response to physical exercise (i.e., physical fatigue). The assessment of physical fatigue with IRT may provide further insights into the mechanisms related to the brain-derived emotion that regulates exercise behaviour.

The unilateral exercise is well-known to induce strength gains on the contralateral limb [23,24,25] via neural adaptations (i.e., cross-over effect). Hence, unilateral exercise may be suitable for discriminating the effect of exercise on activated muscles, nonactivated contralateral muscles, and brain-derived fatigue associated with exercise. Moreover, unilateral exercise limits whole-body thermoregulatory response [26], thus minimizing systemic effects that may affect the proper detection of the exercise’s effect in different body regions.

Therefore, this study intended to investigate the capability of IRT to assess the central and peripheral physiological effect of physical fatigue by measuring facial and muscle temperature modulations in response to a unilateral resistance exercise of the lower limb (i.e., unilateral knee extension exercise). To this aim, skin temperature was measured over four regions of interest (ROIs) placed on the Exercised Leg, Nonexercised Leg, Nose Tip and Corrugator. The ROI located over the exercised muscle was indicative of the variation in microcirculation induced by the exercise in the active muscles. The one placed over the contralateral leg was used to study the cross-over effect, whereas the facial regions were considered indicative of brain-derived fatigue associated with exercise [27]. The thermal effect over different ROIs was studied, employing both time- (i.e., pre–post temperature variations) and frequency-domain approaches. Moreover, the study also aimed to investigate the association between perception of fatigue and temperature response of each ROI, by a correlation analysis between RPE and thermal features evaluated (i.e., skin temperature variations and hemodynamic frequency components).

## 2. Materials and Methods

### 2.1. Participants

The study sample was composed of 8 young healthy female subjects (mean ± standard deviation, age: 24.8 ± 4.9 years; mass: 63.3 ± 7.2 kg; stature: 164 ± 4.7 cm), recruited from a subelite futsal team. Inclusion criteria were no history of musculoskeletal injury in the lower limbs within the year before the study and a training routine based on resistance exercise (apart from sport-specific futsal training) of at least 1 session per week. Exclusion criteria consisted of altered fitness status, which refers to disease or illness presence. None of the participants reported recent lower limb injuries at the time of data collection.

### 2.2. Experimental Protocol

**Figure 1:**
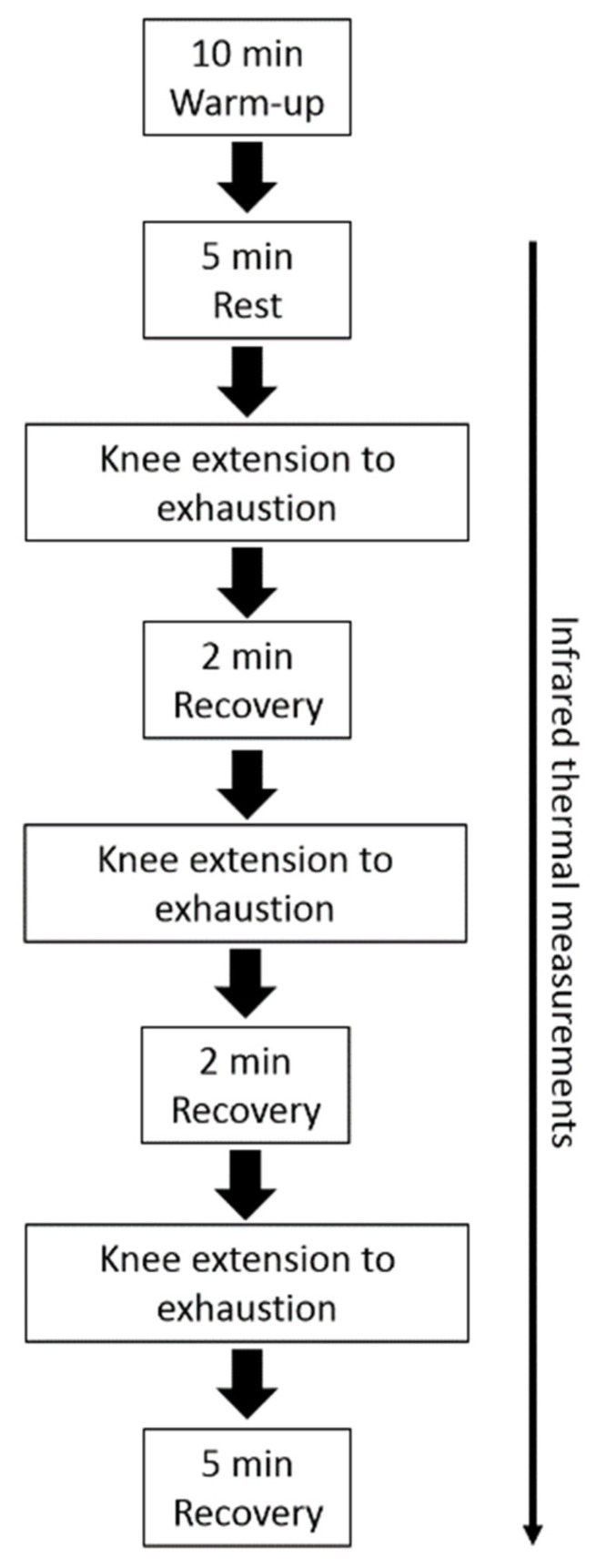
Schematic representation of the experimental session.

Participants were instructed to not perform strenuous physical activity in the two days before the trials, and to avoid alcoholic or caffeine-containing products and creams or cosmetics for four hours before the start of the experiment [28]. All the experimental sessions were performed in the late morning to reduce possible effects induced by the circadian rhythm variations. A preliminary session was provided in order to collect anthropometric measurements, to establish the load of maximal repetition (1 RM) in knee extension of the dominant leg, and also to allow participants to familiarize with the experimental procedures. All the participants were right-leg-dominant. Participants warmed up by completing a number of submaximal repetitions (~15) at approximately 20% of the perceived 1 RM. After 60 s of rest, an initial weight within the subject’s perceived capacity (~50–70%) was chosen. Resistance was progressively increased by 10.0–20.0% from the previous successful attempt until the participants could not complete a single repetition throughout the full range of motion. The range of motion was set to 105° (i.e., from starting position of 75° to ending position of 180°). The 1 RM was determined as the maximum weight participants were able to lift once. A rest period of 300 s was given between each trial, and the 1 RM was obtained within five trials. In the experimental session, participants performed a standardized warmup, i.e., 10 min of walking on a treadmill. After that, and before the beginning of the exercises, participants acclimated to the room conditions (temperature 22–24 °C; relative humidity 50 ± 5%; no direct ventilation and constant intensity of light) for 5 min, at rest, remaining seated on the knee extension machine (Teca srl, Ortona, Italy). Then, participants underwent the knee extension exercise using an intensity of ~50% of 1 RM. The exercise session consisted of three sets of knee extensions with an interset rest period of 3 min. Participants were asked to repeat the movement until exhaustion (concentric failure) following the 1 s pace for each phase of contraction [29]. Shoulder straps were used to stabilize the participants and to minimize the use of trunk muscles during the exercises. At the end of each exercise set, participants were asked to provide RPE, expressed as a number between 1 and 10 [30]. All participants were familiar with the use of RPE as part of their regular training load monitoring process. The protocol of the experimental session is shown in Figure 1.

### 2.3. Thermal Imaging Measurements

**Figure 2:**
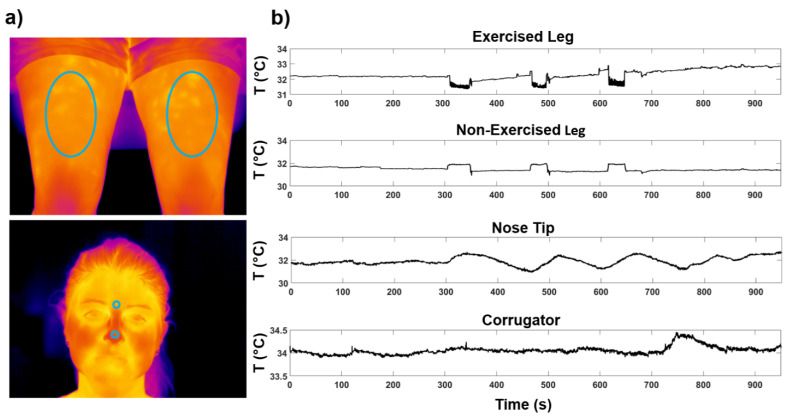
(**a**) ROIs placed over a representative participant; (**b**) temperature time courses of each ROI during the experiment.

Two digital thermal infrared cameras FLIR SC660 (640 × 480 bolometer FPA, sensitivity/Noise Equivalent Temperature difference: <30 mK @ 30 °C, FOV: 24° × 18°) were used in this study. The cameras were placed at 60 cm from the participant and pointed one towards the face and the other towards the legs of the subject (Figure 2). The sample frequency was 10 Hz. To remove the effects related to the potential drift/shift of the sensor’s response and optical artifacts, the camera was blackbody calibrated. Since the participant could freely move during the experiment, the quality of all the recorded thermal videos was preventively checked by visual inspection. No video was rejected. Four ROIs were selected: Exercised Leg, Nonexercised Leg, Nose Tip and Corrugator (Figure 2a).

### 2.4. Thermal Imaging Data Analysis

The mean value of the pixels of each ROI was extracted for each frame (T_ROI_) in order to obtain the temperature dynamic for the duration of the experiment. To this aim, a tracking algorithm was used to track each ROI across the images of the video to properly consider the temperature from each thermogram. The tracking software has been developed and validated in Perpetuini et al. (2021) [31]. Both time-domain and frequency-domain data analyses were performed on the temperature time courses. Concerning the time-domain analysis, the average temperature variation (∆T_ROI_) between the last recovery and the initial baseline temperature was considered for each ROI. Specifically, T_ROI_ was averaged over a temporal window of 10 s (i.e., from 20 s to 10 s before the first repetition and from 10 s before the end of the experiment). Regarding the frequency-domain analysis, the power spectral density (PSD) of the thermal time course was computed. The area under the curve of the PSD was evaluated for different frequency bands [32]: metabolic band (0.003–0.02 Hz) associated with microvascular activity, neurogenic band (0.02–0.04 Hz) related to intrinsic neuronal activity, myogenic band (0.04–0.15 Hz) associated with activity of smooth muscles of arterioles, respiratory band (0.15–0.5 Hz) indicative of the breathing function, and cardiac band (0.5–1 Hz) suggestive of the heart function. Particularly, in order to avoid the effect of motion artifacts and to investigate temporal windows larger than the temporal oscillations associated with the frequency bands, a time window of 5 min before and after the experiment was selected to compute the PSD. Finally, the variation in PSD (∆PSD) between the end and the start of the experiment was calculated. Data were processed using MATLAB 2016b© (The Mathworks Inc., Natick, MA, USA).

### 2.5. Statistical Analysis

Data were expressed as mean ± standard deviation (SD). The normality of the data distribution was checked by the Shapiro–Wilk’s normality test. All the data met the assumption of normality. A one-way analysis of variance with repeated measures (ANOVA RM) was used to compare the effect of the exercise on the thermal features between the ROIs [33]. Least significant difference (LSD) post hoc analyses were used to compare pairs of means. A correlation analysis between RPE and the thermal features obtained by both time- and frequency-domain analysis was performed using the r Pearson’s correlation coefficient. The statistical analysis was performed using Graphpad Prism software (version 7.0, Graphpad, San Diego, CA, USA). A *p*-value lower than 0.05 was considered statistically significant.

## 3. Results

**Figure 3:**
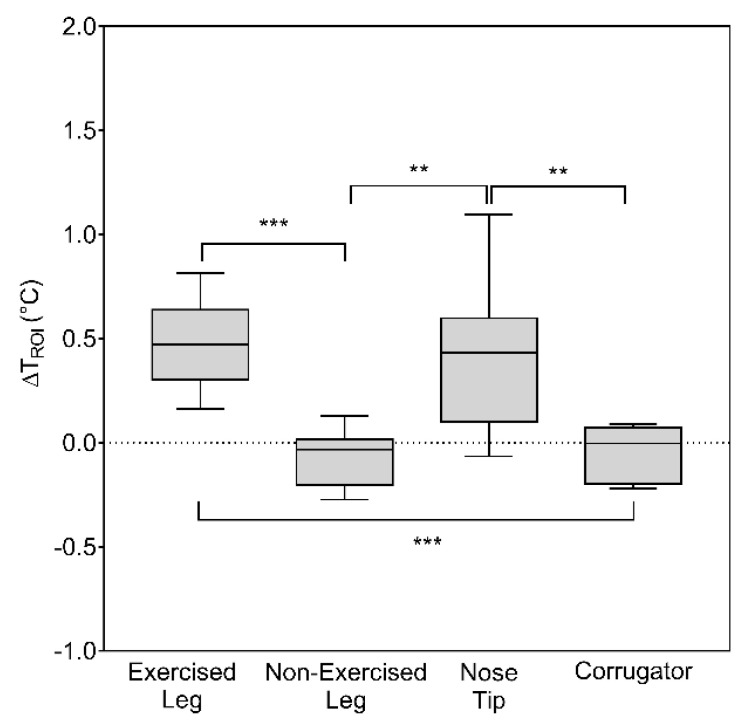
Effect of knee extension exercise on ∆T_ROI_ of Exercised Leg, Nonexercised Leg, Nose Tip, and Corrugator. Each box shows the median and interquartile range, with the whiskers indicating the range of values. ** *p* < 0.01; *** *p* < 0.001 for pairwise comparisons between ROIs.

The temperature time course of the different ROIs of a representative participant is shown in Figure 2b. The ANOVA RM revealed a significant difference in ∆T_ROI_ between the different ROIs (F_(1.41,9.81)_ = 15.14; *p* = 0.0018). Figure 3 reports the boxplots of ∆T_ROI_ of each ROI, together with the post hoc results.

**Figure 4:**
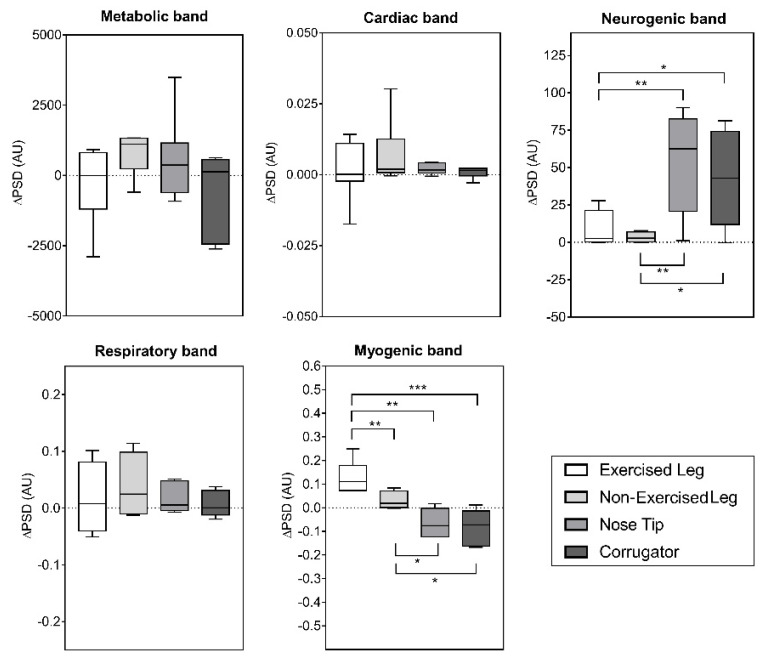
Effect of knee extension exercise on ∆PSD of frequency bands of Exercised Leg, Nonexercised Leg, Nose Tip, and Corrugator. The box shows the median and interquartile range with the whiskers indicating the range of values. * *p* < 0.05; ** *p* < 0.01; *** *p* < 0.001 for pairwise comparisons between ROIs.

Concerning the frequency-domain analysis, the ANOVA RM did not reveal significant differences between the different ROIs in ∆PSD of the metabolic, cardiac and respiratory bands. Conversely, there was a significant difference between ROIs in ∆PSD of myogenic (F_(1.34,9.39)_ = 15.20; *p* = 0.0021) and neurogenic bands (F_(1.75,12.26)_ = 9.96; *p* = 0.0034). The boxplots showing ∆PSD for all the frequency bands are shown in Figure 4, together with the post hoc results.

The r Pearson’s correlation coefficients between RPE and the thermal features evaluated (i.e., ∆T_ROI_ and ∆PSD) are reported in Table 1. Particularly, a negative significant correlation was found between RPE and ∆T_ROI_ of the Exercised Leg and ∆PSD of the myogenic band computed for the Nose Tip and the Corrugator. Moreover, a positive significant correlation was found between RPE and ∆PSD of the neurogenic band calculated for the Nose Tip.

## 4. Discussion

The aim of the present study was to investigate the capability of IRT to assess the central and peripheral physiological effects of exercise. Specifically, a unilateral resistance exercise was used in order to investigate the effect of the exercise on the facial (i.e., Nose Tip and Corrugator) and muscles (i.e., Exercised and Nonexercised Legs) skin temperature time courses. To the best of the authors’ knowledge, this is the first study combining a time- and a frequency-domain analysis on thermographic data to assess the central and peripheral effects of fatiguing exercise.

Time-domain analysis showed an increment in the skin temperature of the Exercised Leg and Nose Tip, whereas a slight decrement in the other ROIs (i.e., Nonexercised Leg and Corrugator) was found (Figure 3). These findings are in line with a previous study by Escamilla-Galindo et al. (2017), showing the same trend for the skin temperature variation of exercised and nonexercised muscles during a unilateral resistance exercise [26]. In fact, their main results showed that, independently of the limb, a modification of the skin temperature was found in response to unilateral exercise [26]. The hypothesis that exercise was performed to exhaustion was corroborated by the significant correlation between RPE and ∆T_ROI_ of the Exercised Leg. Moreover, the significant variation of ∆T_ROI_ on the Nose Tip found in the present study could be suggestive of the effect of exercise-induced fatigue on facial temperature. In fact, it is well-known that the facial temperature variations and distribution could be indicative of physical fatigue [34]. Notably, facial temperature is a proxy of the psychophysiological and emotional state of the subjects [5]. As fatigue is a brain-derived emotion [16], the facial temperature variations observed in response to fatiguing exercise may be related to the perception of fatigue [34]. It is worth noticing that ∆T_ROI_ was calculated based on the difference between the end of the recovery phase and the previous seconds before the first resistance exercise set. Hence, such a metric is sensitive to a cumulative effect of both exercise and the recovery phase, characterized by an initial vasoconstriction followed by a vasodilation, respectively [12]. However, the effectiveness of other metrics indicative of the skin temperature response to exercised limb, and of different statistical indices describing skin temperature distribution, should be further explored within the same experimental paradigm [35].

The results of the frequency-domain analysis showed a significant higher power content in the neurogenic band for the Corrugator and Nose Tip with respect to the muscle ROIs (Exercised and Nonexercised Legs), as shown in Figure 4. The neurogenic band is associated with nerve activation [36], hence it may be possible that the higher activity for this band in the facial ROIs could reflect the state of fatigue. This hypothesis was further corroborated by the significant correlation between RPE and the power content of this band for the Nose Tip (Table 1). Interestingly, a significant negative correlation between RPE and the power content of the myogenic band for the Corrugator and Nose Tip were found (Table 1). A possible explanation for these findings may be related to the physiological functions associated with these frequency bands. In fact, the myogenic band is related with the endothelial activity [37,38]: the endothelium forms an interface between the blood circulating in the lumen and the vessel wall, helping to control the flow of substances and fluid into and out of a tissue. The negative correlation between RPE and ∆PSD of the myogenic band could be ascribed to a modulation of substance transport in the blood stream related to the autonomic activity [38]. Indeed, the autonomic nervous system is considered one of the key factors modulating the behaviour of the endothelial functions [39]. Furthermore, endothelial cells interact with various circulating factors in the blood stream and react to these changes to maintain homeostasis. These notions can contribute to explain, at least partially, the significant correlation revealed between RPE and myogenic bands for the facial ROIs.

Another aspect that is worth discussing is the effect of the unilateral knee extension exercise on the contralateral leg. Specifically, higher myogenic frequency contents were found in the Exercised Leg with respect to the Nonexercised Leg (Figure 4). The higher power of the myogenic frequency band of the Exercised-Leg with respect to the contralateral one may be related to an increment in the myogenic activity in response to the exercise. In fact, it is well-known that a possible mechanism related to the increment in strength in the contralateral leg after a unilateral resistance training relies on the release of vasodilators in response to exercise [24,40]. However, the size of the effect of strength increment in the contralateral limb when exercising unilaterally was reported as small [23,40]. Indeed, further studies investigating the possible mechanisms related to the cross-over effect could be warranted.

The present study has some limitations that should be acknowledged. First, the relatively low sample size might have contributed to impair statistical power to highlight possible differences between the ROIs. Although the reduced sample size would not allow a generalization of these results, it should be highlighted that the adopted design along with the homogeneous sample (subelite female futsal players) would partially compensate for that limitation by also reducing data variability. However, increasing the number of participants, and extending measurements on male individuals, may provide more generalizable results [41]. A larger sample size would allow to employ methods of machine learning and artificial intelligence to assess the state of fatigue of the participants [34]. Moreover, it could be interesting to develop a multimodal approach to further validate the performance of IRT for assessing physical fatigue. The lack of other physiological measurements (such as muscle activation, and core and muscle temperature) might not have permitted a deep understanding of the underlying mechanisms in response to unilateral resistance exercise to fatigue. For instance, further studies should focus on combining IRT with electromyography in order to investigate the relationship between skin temperature and muscle activation in response to fatiguing exercise. Moreover, information on core and muscle temperature may further contribute to provide a complete picture of the whole thermoregulatory process related to fatigue.

Overall, although preliminary, these results demonstrated the capability of IRT to assess the state of physical fatigue in response to a resistance training session. It is worth noticing that the resistance training session considered in the present study involved three sets of knee extension exercise, as commonly employed within a field-based training routine. However, further studies should investigate the capability of IRT to assess exercise-induced fatigue also in other kind of resistance training involving different numbers of sets and repetitions. From a practical viewpoint, thanks to the contactless features of this technique, IRT could be a powerful tool for the evaluation of the psychophysiological state of the subjects and further monitoring resistance exercises within a strength training programme [42]. For instance, these findings could pave the way to the employment of IRT within the resistance training routine, in the field of training load monitoring process, in accordance with the psychophysiological conditions of the athletes. Researchers and practitioners in the sports field may take advantage of this technique thanks to its noninvasive, contactless, and relatively low-cost features.

## 5. Conclusions

The findings of the present study demonstrate the capabilities of IRT to assess physical fatigue during a unilateral resistance exercise. Specifically, differences in temperature variations were found between different ROIs, revealing the potentiality of thermal imaging to discriminate physiological processes underlying the perception of fatigue. These results were also corroborated by the frequency-domain analysis, showing different behaviours of ROIs in the neurogenic and myogenic bands. Finally, the significant correlations between thermal features (i.e., ∆PSD of myogenic and neurogenic bands of the facial areas, and ∆T_ROI_ of the Exercised Leg) and RPE confirmed the ability of IRT based on both time- and frequency-domain analyses to assess both peripheral and central effects of exercise to fatigue. Although preliminary, these findings could foster the application of this technology within the field of training load monitoring process.

## Figures and Tables

**Table 1:** The r Pearson’s correlation coefficients between RPE and the thermal features for each ROI. * *p* < 0.05, ** *p* < 0.01.

	Exercised Leg	Nonexercised Leg	Nose Tip	Corrugator
RPE vs. ∆T_ROI_	−0.84 **	−0.21	−0.16	−0.39
RPE vs. ∆PSD Metabolic	0.54	0.49	−0.52	−0.43
RPE vs. ∆PSD Cardiac	0.20	0.41	0.42	0.57
RPE vs. ∆PSD Respiratory	0.30	0.32	0.26	0.44
RPE vs. ∆PSD Neurogenic	0.51	0.51	0.75 *	0.29
RPE vs. ∆PSD Myogenic	−0.41	−0.22	−0.71 *	−0.80 *

## Data Availability

The data presented in this study are available on request from the corresponding author. The data are not publicly available due to privacy issues.
